# Vertically stratified microbial diversity and keystone species driving element cycling in the Magellan seamount sediments

**DOI:** 10.1099/mgen.0.001493

**Published:** 2025-12-05

**Authors:** Chengcheng Li, Huameng Ge, Wenhao Huang, Dewi Seswita Zilda, Ocky Karna Radjasa, Linlin Zhao, Bailin Cong, Shenghao Liu, Zhaohui Zhang

**Affiliations:** 1 Marine Ecology Research Center, First Institute of Oceanography, Ministry of Natural Resources, Qingdao 266061, PR China; 2 Laboratory for Marine Ecology and Environmental Science, Qingdao Marine Science and Technology Center, Qingdao 266237, PR China; 3 Research Center for Deep Sea, Earth Sciences and Maritime Research Organization, National Research and Innovation Agency (BRIN), JL Pasir Putih Raya, Pademangan, North Jakarta City, Jakarta, 14430, Indonesia

**Keywords:** biogeochemical cycling, co-occurrence network, deep-sea sediment, Magellan seamounts, metagenomics, microbial community, polymetallic nodules, vertical distribution

## Abstract

Deep-sea polymetallic nodules, rich in cobalt, nickel and titanium, are valuable for electronics, aerospace and energy industries. However, the vertical distribution and ecological functions of prokaryotic communities in sediments beneath nodules from the Magellan seamounts, a unique microbial habitat characterized by ultra-slow sedimentation rates (0.4–4 mm ky^−1^) and heterogeneous metal gradients, remain poorly characterized. In our research, 16S rRNA gene amplicon sequencing and metagenomic analyses of sediment cores (0–20 cm) from the western Pacific polymetallic nodule province revealed statistically significant decreases in prokaryotic diversity (Shannon index: 9.446 to 2.288; *P*<0.001). *Proteobacteria*, *Crenarchaeota*, *Chloroflexi* and *Bacteroidota* were the dominant taxa. The microbial co-occurrence network in the surface layer had a longer mean path length (2.11 vs 1 in the bottom layer) and a larger network diameter (11 vs 1), indicating a loose community structure and greater resistance to disturbance, while the bottom microbial network had a higher density (0.037 vs 0.01) and clustering coefficient (0.32 vs 1), suggesting tight microbial interactions. The concentrations of MnO (6.96–9.41 µg g^−1^) and P₂O₅ (2.55–3.89 µg g^−1^) gradually decreased with increasing depth. The concentrations of Co and Pb were relatively high in the surface sediments (0–8 cm) but decreased significantly below 8 cm. In contrast, the concentrations of Fe₂O₃ and As increased with depth. The environmental factors depth, MnO, Fe₂O₃ and heavy metals (Cr, Zn and Cu) were found to be the main drivers of the microbial community structure. We assembled 122 metagenome-assembled genomes from the metagenomic data. Gene abundance analysis revealed that sox genes (*soxB*/*C*/*D*/*X*/*Y*/*Z*) and assimilatory sulphate reduction genes (*cysC* and *cysH*) were highly abundant in the surface sediment, whereas the abundance of dissimilatory sulphate reduction genes (*dsrA* and *dsrB*) was enhanced in the bottom layer, reflecting a hierarchical adaptive strategy for sulphur metabolism. Our study expands current knowledge on the vertical variations of microbial diversity and microbially driven biogeochemical cycling in deep-sea settings underneath polymetallic nodules. Characterizing the microbial community underneath those nodules may provide insights into microbial resilience in extreme oligotrophic environments and valuable insights for future deep-sea mining activities.

**Impact Statement**

The deep sea floor contains valuable mineral resources like polymetallic nodules rich in cobalt, nickel and titanium, crucial for electronics, aerospace and energy industries. Micro-organisms are vital primary producers in the deep-sea food chain, essential for ecosystem health and diversity. However, the geographic distribution of microbial communities in western Pacific polymetallic nodule regions, the adaptation of functional microorganisms and their ecological functions remain unknown. This study used 16S rRNA gene amplicon sequencing and metagenomic analysis to explore prokaryotic communities and biogeochemical cycling beneath deep-sea polymetallic nodules across sediment layers (0–20 cm). The results revealed significant vertical diversity variations, with dominant phyla like *Proteobacteria*, *Crenarchaeota*, *Chloroflexi* and *Bacteroidota*. Depth primarily influenced prokaryotic distribution, while elements such as P_2_O_5_, Fe_2_O_3_ and MnO and heavy metals shaped community patterns. By identifying 122 metagenome-assembled genomes, we compared microbial cycling processes of various elements and their gene abundance. This study contributes to the comparative analysis of microbial adaptation processes in diverse western Pacific habitats, offering a baseline for monitoring the environmental impacts of deep-sea mining and developing ecological restoration strategies.

## Data Availability

The raw sequencing data generated in this study have been deposited in the NCBI Sequence Read Archive (SRA) under BioProject accession number PRJNA1365450 (SRA run accessions: SRR36115654–SRR36115663). The data are publicly available as of the date of this submission.

## Introduction

The deep sea, the largest marine ecosystem, is generally divided into hydrothermal vents, cold seeps, seamounts, ocean trenches, abyssal plains and mid-ocean ridges. It is characterized by lack of light, low temperature, high pressure and oligotrophic conditions, making it an extreme environment [1]. The deep sea harbours abundant and diverse microbial communities [2], which drive the synthesis, transformation and breakdown of organic and inorganic compounds through unique metabolic pathways, including chemosynthesis and heterotrophic decomposition. These chemosynthetic organisms act as primary producers, continuously supplying material and energy to marine ecosystems [3]. Their ability to utilize metals as energy sources also makes them essential to the biogeochemical cycling of elements in deep-sea sediments [4,5].

Hydrothermal vent micro-organisms such as *Hydrogenovibrio* cope with extreme dynamic environments through metabolic plasticity (concurrent utilization of sulphur/hydrogen/iron as electron donors) and novel resistance pathways (e.g. uncharacterized iron-oxidation routes). Their ultrahigh thiosulphate oxidation rate (952 mmol h^−1^) and robust CO_2_ fixation capacity directly drive *in situ* element cycling [6]. In sediments containing ferromanganese nodules, microbes have evolved specific adaptations to survive and thrive in extreme environments. These adaptations include unique membrane compositions, stress response proteins, specialized enzymes (e.g. for metal redox reactions) and metabolic versatility, allowing them to survive and adapt to extreme environments by obtaining energy through metal ion redox reactions [7]. Exploring microbial diversity and interaction patterns is critical for revealing the vertical succession of complex microbial communities in marine sediments [8]. The western Pacific Ocean, with its metal-rich sedimentary environment, provides an ideal model system for studying microbially mediated element cycles.

Among diverse deep-sea ecosystems, the western Pacific Ocean represents a critical region for studying microbial biogeography due to its unique geological and ecological features. It is a global marine biodiversity hotspot [9], hosting exceptionally rich and taxonomically diverse ecosystems [10]. The Magellan seamounts, located in the Northwest Pacific Plate, form a geologically complex region characterized by heterogeneous distributions of intermountain basins, isolated seamounts and seamount chains [11]. The Magellan seamounts have a lot of manganese nodules, which are strategically valuable due to high concentrations of industrially essential metals such as nickel, copper, cobalt and manganese [12,13]. The redox interfaces created by polymetallic nodules enable micro-organisms to serve as central engines of elemental cycling through metal/sulphur redox reactions, such as Mn^2+^/Mn^4+^ and S^2-^/SO_4_^2-^ transformations. Microbial communities play vital roles in biogeochemical cycling within these environments. The Magellan seamount cluster has a sedimentation rate of 0.4–4 mm Ky^−1^ [14], indicating that the microbial community changes with increasing burial depth. In seamount sediments of the Southwest Pacific Ocean, microbial communities of seamount sediments have significantly higher richness levels and diversities of the microbial communities compared to those in sediments from cold seeps and ocean trenches, and *Halomonas* is the dominant microbial species [15]. At Loihi seamount, microbial communities are dominated by *α*-*Proteobacteria*, *γ*-*Proteobacteria* and *Thaumarchaeota*, driving metabolic processes such as carbon sequestration, denitrification, dissimilatory sulphate reduction and sulphur oxidation [16]. Microbial distribution was mostly governed by depth, but heavy metals (such as iron, copper, nickel, cobalt and zinc) can also have a major impact on their distribution [17]. However, due to sampling difficulties and the challenges of culturing deep-sea microbes in laboratories, the microbial diversity, community interaction along environmental gradients and microbially driven biogeochemical cycling in Magellan seamounts are still far from fully understood [18,19]. To provide a baseline reference for the protection of marine ecosystems, more research is necessary to enhance our knowledge of the microbial community structures and their environmental responses in deep-sea extreme environments, especially for areas rich in polymetallic nodules [20].

In our research, sediment samples were collected from the Magellan seamount at various depths (0–20 cm) in the western Pacific Ocean. Given the documented significance of metal oxides (e.g. Fe and Mn) in microbial energy metabolism within polymetallic nodule environments [7,17] and the fundamental roles of carbon, nitrogen and sulphur in microbial biomass, energy flow and biogeochemical cycling [3,5,16], we focused our geochemical analyses on total iron (Fe₂O₃), manganese (MnO), total sulphur (TS), total organic carbon (TOC), total nitrogen (TN) and total carbon (TC), alongside key heavy metals (Cu, Zn, Cr, Co, Pb and As). Based on 16S rRNA gene sequencing, we revealed prokaryotic diversity and community compositions across different depth layers and their co-occurrence network relationships. High-depth metagenomic sequencing was used to obtain the dominant microbial genomes and key gene sequences encoding metabolic pathways. Meanwhile, we also estimated the contribution of physicochemical parameters in shaping the microbial community and microbially driven biogeochemical cycling. Our results provide a new understanding on the vertical distribution and environmental adaptation strategies of microbes underneath polymetallic nodules in the Magellan seamount sediments.

## Methods

### Site description and sampling

The sediment core was collected at a water depth of ~5,500 m (153.11° E 19.42 °N) using a stainless-steel box corer (sampling area: 0.25 m²) during the September 2023 expedition of the R/V Shenhai-1 at the Magellan seamount in the western Pacific Ocean. After retrieval, overlying water was carefully removed, and a Polyvinyl chloride (PVC) tube (inner diameter: 20 cm) was inserted into the centre of the box corer to obtain an intact sediment core. The core was then vertically divided into two halves along its central axis. One half of the PVC tube was subsampled using a stainless cutter at fine intervals (0–1, 1–2, 2–3, 3–4, 4–5, 5–8, 8–11, 11–14, 14–17 and 17–20 cm) for metagenomic sequencing, while the other half was subsampled at broader intervals (0–2, 2–5, 5–8, 8–11, 11–14, 14–17 and 17–20 cm for 16S rRNA gene sequencing). Each sediment was immediately transferred to sterile plastic containers and stored at −80 °C until the extraction of DNA.

### Physicochemical parameter analysis

For physicochemical characterization, sediment subsamples were collected from the same half-core intervals as those used for 16S rRNA gene sequencing. These samples were oven-dried for 4 h at 105 °C. We accurately weighed 50 mg of the sample into a Polytetrafluoroethylene (PTFE) digestion vessel and added 1.5 ml of high-purity HNO_3_ and 1.5 ml of high-purity HF. The above mixture was thoroughly shaken, sealed with a lid, placed in a steel sleeve and heated in an oven at 190 °C for 48 h to dissolve. After cooling at room temperature (25 °C), the digestion vessel was placed on a hot plate at 105 °C and evaporated to near dryness (until only a thin film of residue remained). Then 1 ml of high-purity HNO_3_ was added and evaporated again to complete dryness. Following this, 3 ml of 50 % HNO_3_ and 1 p.p.m. Rh internal standard solution (0.5 ml) were added. The vessel was sealed with a lid, placed in a steel sleeve and heated in an oven at 150 °C for 8 h to dissolve. After cooling, the digestion vessel was removed and diluted to 50 g with deionized water. Fe_2_O_3_, MnO, P_2_O_5_ and TS were measured using an inductively coupled plasma 0ptical emission spectrometer produced by Thermo Fisher Scientific (Waltham, MA, USA) (iCAP7000). We took out 10 g from the above test solution, diluted to 20 g with deionized water and measured copper, zinc, Cr, Co and Pb element contents using an inductively coupled plasma mass spectrometer (iCAPRQ model). The contents of TOC and TN in sediment were measured using the elemental analyser (German, Vario EL III). The preliminary samples were dried in an oven at 60 °C for 2 h. Subsequently, the 1 g dried sample was weighed and transferred to a polypropylene test tube. Following this step, 2 ml 5 mol l^−1^ hydrochloric acid solution was added, and the test tube was sonicated in an ultrasonic cleaner for 3 h to remove inorganic carbon. During this process, we added hydrochloric acid solution several times (2 ml each time) and vortexed until no bubbles were produced in the sample. We then added 50 ml of water, vortexed thoroughly and centrifuged at 4,000 r.p.m. for 10 min. We decanted the supernatant, washed it 2–4 times with distilled water (each time using ~10 ml of water per wash) until the supernatant was neutral, decanted it again, dried it at 60 °C, weighed it accurately to 0.01 g and homogenized the sample. We weighed out 20 mg, wrapped it in a tin boat and analysed the TN, TC and TOC content using the elemental analyser. We accurately weighed 0.5 g of sediment sample and added 10 ml of 1 : 1 aqua regia. The sample was allowed to digest cold at room temperature overnight. It was then heated in a 95 °C water bath for 2 h. After cooling to room temperature, 5 ml of the supernatant was transferred into a polypropylene colorimetric tube. We added 3 ml of thiourea-ascorbic acid, 2.5 ml of 1 mol l^−1^ HCl, brought to a volume of 25 ml, pre-reduced for over half an hour and measured the As content using an atomic fluorescence spectrophotometer (China, Model AFS-930). The total inorganic carbon (TIC) content was calculated by subtracting the TOC content from the TC content.

### DNA extraction and sequencing analysis

DNA was extracted from sediment samples using the Qiagen DNeasy® PowerSoil Pro Kit according to the manufacturer’s instructions (Qiagen, Hilden, Germany). DNA integrity was assessed through agarose gel electrophoresis, while its concentration and purity were determined using a NanoDrop 2000 spectrophotometer (Thermo Fisher Scientific) and Qubit 4 Fluorometer (Thermo Scientific, USA). Amplification was performed using extracted DNA as template, the 515F/806R primer pairs (specific for prokaryotes) [21] and Phusion® High-Fidelity PCR Master Mix (New England Biolabs, Ipswich, MA, USA) in a 30 µl PCR reaction. The PCR thermal cycling regime consisted of an initial denaturation at 98 °C for 60 s, followed by 30 cycles of denaturation at 98 °C for 10 s, annealing at 50 °C for 30 s and extension at 72 °C for 30 s, with a final extension at 72 °C for 5 min. The PCR products were purified using the Agencourt AMPure XP magnetic beads (Beckman Coulter, Brea, CA, USA). Subsequently, the purified products were subjected to library preparation using the NEB Next® Ultra™ II FS DNA PCR-free Library Prep Kit (New England Biolabs) according to the manufacturer’s instructions. The prepared libraries were subjected to sequencing utilizing Illumina NovaSeq 6000 PE250 platform at Novogene Co., Ltd. (Beijing, China). The genomic DNA of seven layers of sediments (three samples per layer) was sequenced based on the 16S rRNA gene to obtain their microbial communities. The reads of each sample were assembled using FLASH (version 1.2.11) [22] software to generate the original data (raw reads). Subsequently, the raw reads were subjected to quality filtering using fastp (version 0.23.1) to remove sequences shorter than 50 bp, with quality values below 20, or containing *N* bases, resulting in filtered data [23].

Subsequently, these reads underwent removal of chimeric sequences by aligning them with the species annotation database (silva version 138.1 database) to detect and eliminate chimaeras, resulting in the generation of the final effective data (effective reads) [24]. The effective reads obtained were denoised using the DADA2 module in QIIME2 software (version QIIME2-202202) to obtain the final Amplicon Sequence Variants (ASVs) and feature table [25]. Species annotation was performed using the silva version 138.1 database [26], a widely accepted and curated reference database for taxonomic classification of bacterial and archaeal 16S rRNA gene sequences, chosen for its comprehensive coverage and accuracy in environmental microbiome studies [27].

### Metagenomic sequencing assembly and annotation

To further characterize the functional potential of sediment microbiomes involved in biogeochemical cycles, metagenomic sequencing was conducted on ten DNA samples from stratified sediments. Illumina PE150 sequencing was performed at Novogene Co., Ltd. The raw reads were filtered using the fastp software to obtain high-quality sequencing data. Metagenome assembly was carried out using MEGAHIT (version 1.1.2) [28], filtering out contigs smaller than 500 bp. The assembly results were evaluated using the QUAST software [29]. Redundancy was reduced using MMseqs2 [30] with a similarity threshold of 95% and coverage threshold of 90%.

Functional gene annotations related to nitrogen, phosphorus, sulphur, iron and plastic degradation enzymes were performed using Diamond (version 0.8.35) against NCycDB [31] (dedicated database for nitrogen cycling genes), SCycDB [32] (dedicated database for sulphur cycling genes), PCycDB [33], Plastic DB [34] and FeGenie [35] (a specialized tool and database designed for the comprehensive annotation of iron metabolism genes in (meta)genomes) databases. Enzymes involved in the degradation, modification or formation of glycosidic bonds were annotated using the Carbohydrate-Active Enzymes (CAZy) database [36] (the standard resource for carbohydrate-active enzyme annotation). Clean data was mapped back to predicted genes using Salmon with default parameters to obtain gene abundances [37]. Contigs from individual metagenomic libraries were binned using MetaWRAP [38], based on tetranucleotide frequency and coverage values, to identify genomes of potential micro-organisms involved in biogeochemical cycles. Subsequently, the bin_refinement module in MetaWRAP was used to retain bins with completeness greater than 70% and contamination levels below 10%. The bins with >90% completeness and <5% contamination were categorized as high-quality bins [39].

Taxonomic annotations were finalized utilizing GTDB-Tk, relying on the Genome Taxonomy Database [40]. The quant_bins module in MetaWRAP was used with default parameters to calculate bin abundances, map clean reads to contigs and compute the length-weighted average coverage of contigs in each bin. Finally, Prodigal version 2.6.3 [41] was utilized with default parameters for gene prediction on the remaining bins.

### Co-occurrence network construction

Microbial co-occurrence networks were constructed and analysed based on the ASVs from the 16S rRNA gene amplicon sequencing. To reduce network complexity, ASVs were selected based on their average relative abundance >0.1% across all 16S rRNA gene amplicon sequencing libraries and presence in over 50% of the samples. The network was built using the ‘Hmisc’ and ‘igraph’ packages in R software (version 4.2.0) and visualized using Gephi software (version: 0.9.2, https://gephi.org/) to depict the relationships among potentially active microbial taxa in the seabed sediment. Pairwise Spearman correlations were computed among the ASVs, with correlation coefficients >0.7 and *P*-values <0.05 (Benjamini and Hochberg-adjusted, BH) considered as valid relationships. To describe the network’s topological properties, metrics such as network size (number of nodes, *N*), network connectivity (total number of connections, *L*), average connectivity (average number of connections per node, average *K*), average strength (average weighted connectivity, average *S*) and average clustering coefficient were calculated.

### Statistical analyses

To assess the alpha diversity of microbial communities, Shannon, Chao1 and Simpson indices were calculated using QIIME2 software. A paired t-test was employed to evaluate significant differences in microbial diversity among three sediment groups. Using R software (version 4.0.1) and the base package ‘vegan’, the overall differences in bacterial and archaeal community compositions were visualized through principal coordinate analysis (PCoA) based on Bray–Curtis distances. Mantel tests were conducted to determine the correlation between sediment and bacterial community structures. Canonical correspondence analysis (CCA) was utilized to analyse the correlation between environmental variables and microbial communities. The ‘ggcor’ package in R was employed to analyse Spearman correlations among environmental factors and their associations with bacterial and archaeal communities.

For indicator species analysis, the top 500 most abundant ASVs from each group were selected. Specificity and occupancy (≥0.7) were calculated and identified using SPEC-OCCU plot analysis [42]. Within-module connectivity (Zi) and among-module connectivity (Pi) thresholds were applied to categorize the ecological functions of individual nodes within the network [43]. The nodes were categorized into four groups based on their characteristics: module hubs (Zi >2.5), network hubs (Zi >2.5 and Pi >0.62), connectors and peripherals (Zi <2.5 and Pi <0.62), and connectors, module hubs and network hubs were theoretically regarded as key taxa in networks [44,45]. Pearson correlation analysis between element cycling gene abundances and geochemical variables was conducted utilizing the ‘LinkET’ and ‘vegan’ packages in R. The gene abundance was visualized with a heatmap.

## Results

### Physicochemical characteristics of sediments at different depths in western Pacific Ocean

The concentrations of Fe₂O₃, MnO, P₂O₅, Cr, Co, Cu, Zn, Pb, As, TOC, TIC, TN and TS were measured to assess their vertical distribution in the sediments of the western Pacific (Fig. S1, available in the online Supplementary Material). The Fe₂O₃ concentrations ranged from 8.4 to 8.77 mg g⁻¹, while As concentrations ranged from 15.93 to 18.02 µg g⁻¹, both showing an increasing trend with depth. MnO content ranged from 0.7 to 0.94 mg g⁻¹, with the highest values observed at 5–8 cm and the lowest values at 8–11 and 11–14 cm. Additionally, TC, TN and TS concentrations gradually decreased with depth. The TC content in the 0–8 cm sediments was about 0.39–0.48%, more than double that in the 17–20 cm sediments. In particular, the concentrations of MnO, P₂O₅, Co, Zn, Pb, TC, TOC and TIC decreased significantly from depths greater than 5–8 cm. These results highlight substantial changes in the environmental characteristics of sediments below 8 cm, suggesting that sediments below this depth provide distinctly different habitats for organisms.

### The diversities and compositions of the microbial community along the sediment depth gradient

**Fig. 1.**
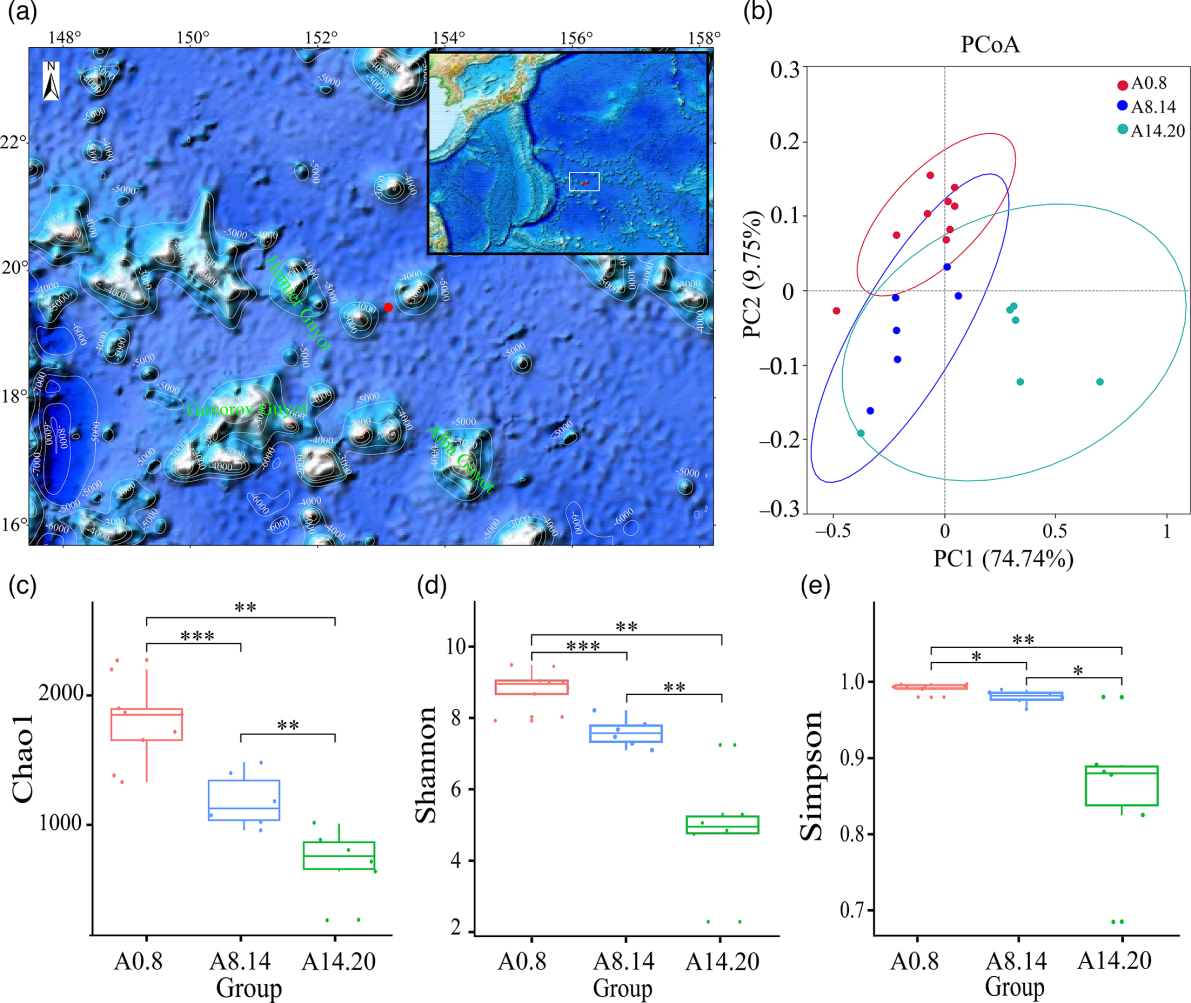
Microbial diversity of sediment samples from the BC07 site in the western Pacific polymetallic nodule area. (a) BC07 sampling site in the western Pacific. (b) PCoA analysis based on Bray–Curtis dissimilarities. (d, e) Prokaryotic microbial Chao1, Shannon and Simpson indices. Asterisks denote significant differences based on one-way ANOVA followed by Tukey HSD (*P<0.05, **P<0.01, ***P<0.001).

Based on 16S rRNA gene amplicon sequencing, we obtained 1,631,910 DNA sequences (assigned to 1,385 archaeal ASVs and 9,378 bacterial ASVs). PCoA revealed three distinct groups of sediment microbial communities (A0-8, A8-14 and A14-20), with the first two principal components accounting for 84.49% of the total variation (PC1 : 74.74% and PC2 : 9.75%) as shown in Fig. 1b. The Chao1, Shannon and Simpson indices of the A0-8 (surface) layer were significantly higher than those of the A8-14 (middle) and A14-20 (bottom) layers (Fig. 1c–e). This indicates a progressive decrease in microbial diversity with increasing sediment depth, highlighting differences in microbial communities at various depths.

**Fig. 2.**
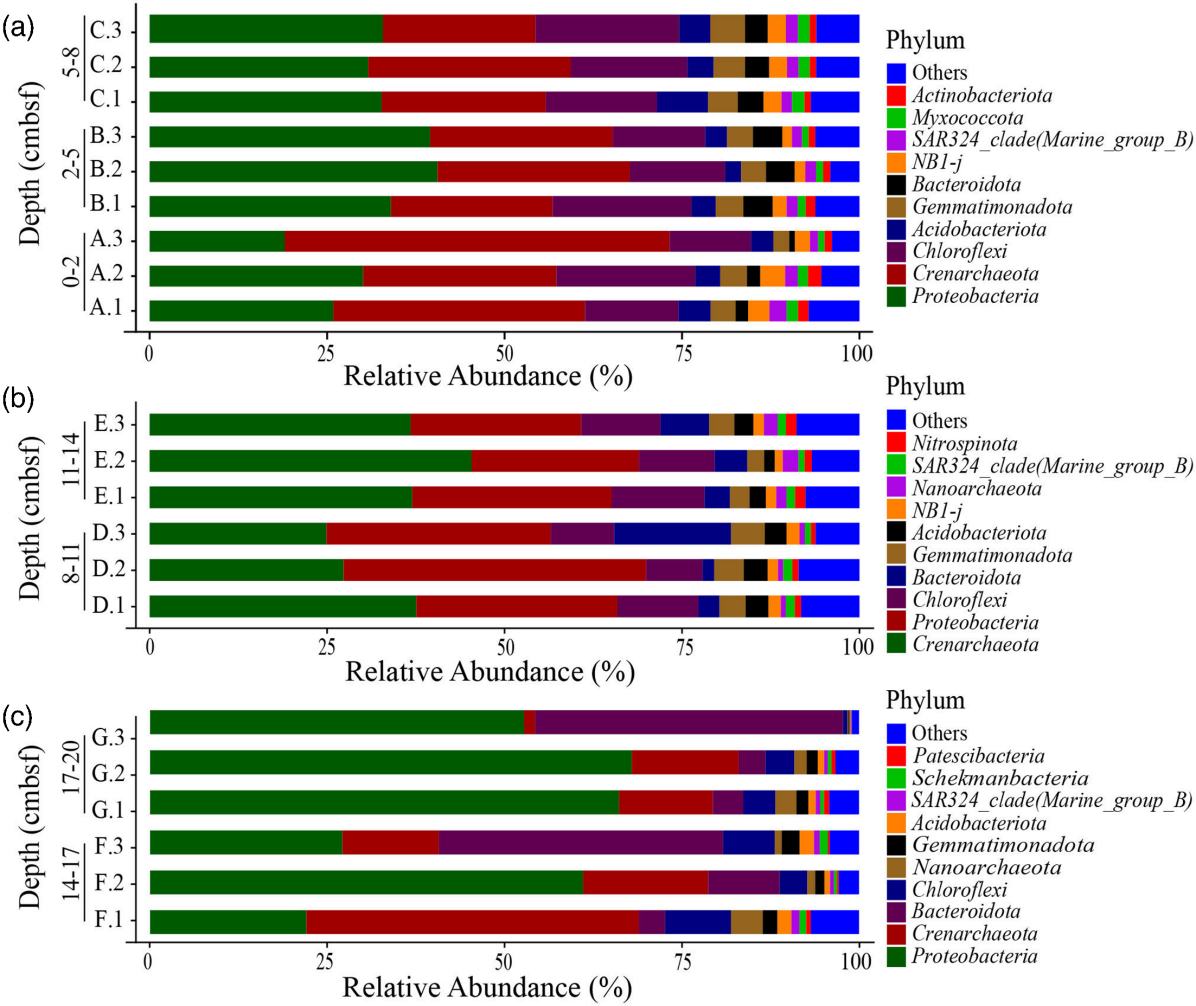
Relative abundance of dominant micro-organisms at phyla in BC07 sediment. Stacked bar plots showing relative abundance of the predominant micro-organisms at the phylum level in (**a**) the surface layer, (**b**) the middle layer and (**c**) the bottom layer of sediment. The cmbsf means cm below the seafloor.

According to the species annotation results, *Proteobacteria*, *Crenarchaeota*, *Chloroflexi* and *Bacteroidota* were the dominant phyla (Fig. 2a–c). *Crenarchaeota* and *Proteobacteria* were identified as the dominant archaeal and bacterial phyla in the 0–8 cm sediment, with relative abundances of 21.5–54.21% and 18.98–40.56%, respectively (Fig. 2a, Table S1). The sediments at depths of 8–14 and 14–20 cm were dominated by *Proteobacteria*, accounting for 23.56–66.17% of the microbial communities in these sediments (Fig. 2b, c). The relative abundance of *Crenarchaeota* significantly decreased in the 14–20 cm sediment layer. At the genus level, *Nitrospina*, *Nitrospira* and *Candidatus_Nitrosopumilus*, which are involved in the nitrogen cycle, were the dominant genera. The relative abundance of *Nitrospira* was highest in the surface layer (0.90%), with lower abundances in the middle (0.85%) and bottom layers (0.29%) (Fig. S2). As a known ammonia-oxidizing genus, *Nitrospira*’s distribution aligns with TN availability (Fig. 5b), confirming its role in nitrogen cycling. We speculated that this may be significantly correlated with the decrease in TN content with depth. Additionally, the predominant genera also included *Woeseia*, *Chryseobacterium* and *Ralstonia. Woeseia* had higher abundance at 0–8 cm compared to 8–14 and 14–20 cm. This chemolithoautotroph utilizes inorganic sulphur compounds for energy, explaining its correlation with TS/TC (Fig. 5b). In contrast, *Ralstonia*, also from the phylum *Proteobacteria*, showed lower abundance at 0–8 cm compared to the deeper layers of 8–14 and 14–20 cm (Fig. S2).

### Co-occurrence networks of the microbial communities in different sediment layers

**Fig. 3.**
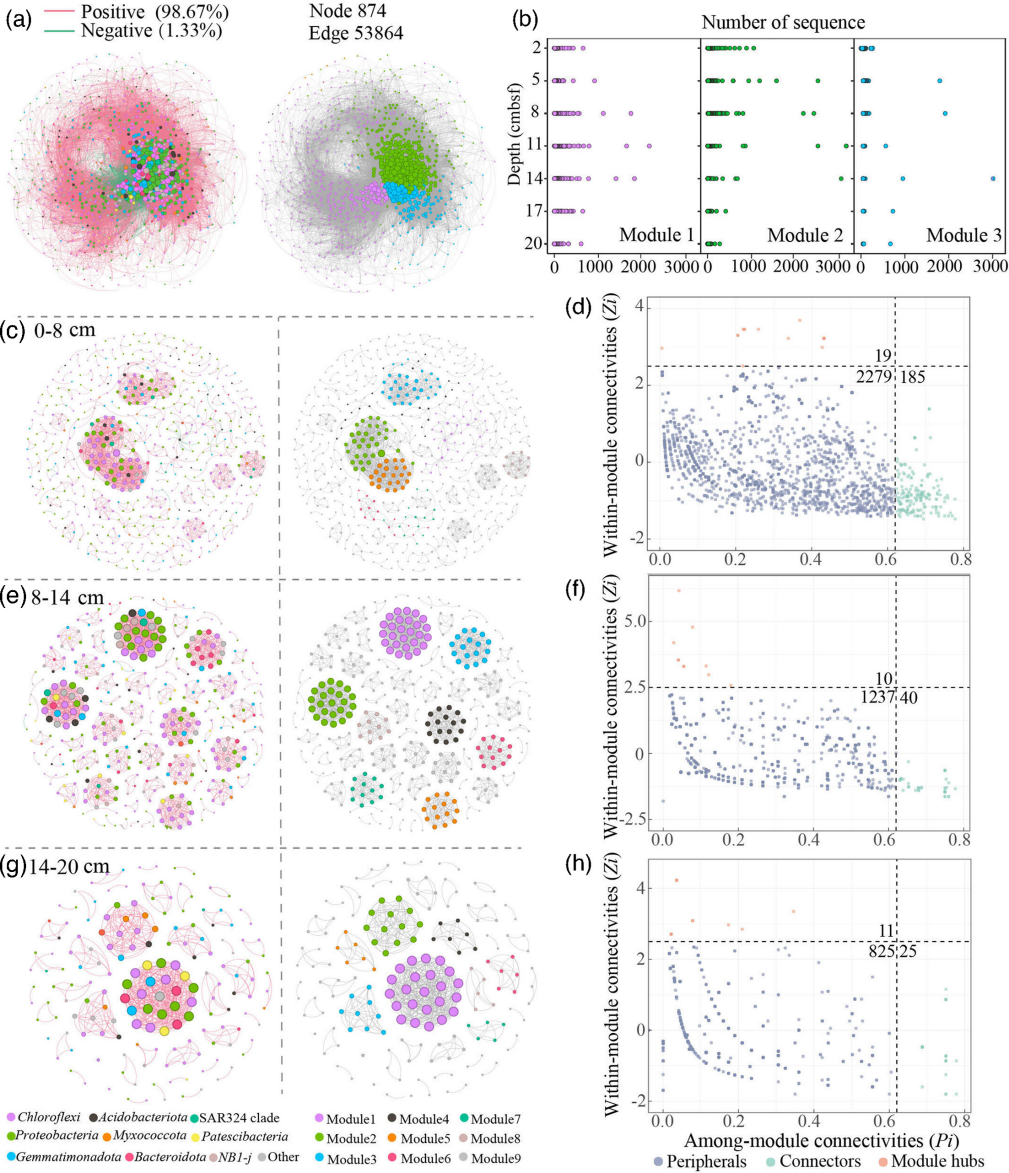
Co-occurrence networks and Zi-Pi plots with ASVs of prokaryotic microorganism communities. (**a, b**) Microbial co-occurrence network analysis of all samples at the phylum level and module structure, along with the sequence counts of each ASV in specific depths within different modules. (**c, e, g**) Co-occurrence network analyses at phylum and module structure from different groups. (**d, f, h**) Zi-Pi plots of ASVs from micro-organism communities within networks. In the network, connections represent a strong (*r*>|0.7|) and significant (*P*<0.05) correlation between two nodes. Each node is coloured for a phylum. The node size is proportional to the number of connections with other nodes (degree). Nodes are connected by pink edges for positive interactions and green edges for negative interactions. The threshold values for categorizing ASVs based on Zi and Pi were 2.5 and 0.62, respectively.

To determine changes of the co-occurrence patterns along a depth gradient, we conducted co-occurrence network analysis on samples collected from a spatial transect at various depths (Fig. 3). The complete network comprised 1,468 nodes connected by 158,298 edges (Table S2). ASVs annotated to *Proteobacteria* (31.99%), *Chloroflexi* (30.95%) and *Gemmatimonadota* (9.01%) phyla showed extensive correlations with other ASVs throughout the network. Notably, the positive edges occupied 98.67% of the total connections, suggesting widespread functional dependencies. We hypothesize that this cooperative pattern reflects metabolic cross-feeding [e.g. sulphur-oxidizing bacteria (SOB) providing electron donors for nitrate reducers] as an adaptive strategy in nutrient-limited sediments. We found that 98.51% nodes in networks were assigned to three modules (modules 1, 2 and 3), accounting for 42.79%, 40.27% and 15.45% of the whole network, respectively (Fig. 3a). The majority of ASVs in module 1 had higher relative abundances at depths of 8–14 cm, while those in module 2 were more abundant at 0–8 cm (Fig. 3b). In module 1 (8–14 cm dominant), the co-dominance of *Gemmatimonadota* (organic matter degrader) and sulphate-reducing *Proteobacteria* (e.g. *dsrAB* carriers in Fig. 7) suggests potential syntrophy: the former may release H_2_ during fermentation, fuelling the latter’s dissimilatory sulphate reduction.

To explore how microbial co-occurrence patterns varied with depth, we analysed separate co-occurrence networks, and the topological characteristics were analysed for samples from 0 to 8, 8–14 and 14–20 cm (Fig. 3c, e and g, Table S2). The nodes (874), edges (53,863), average degree (123.25) and density (0.141) of the whole network were higher than those in separate co-occurrence networks, indicating the whole network was the most robust (Table S2). Additionally, the 0–8 cm network exhibited a greater average path length and network diameter than the 8–14 and 14–20 cm networks. Together with the significantly higher *α*-diversity observed in the top community compared to the deeper layers (Fig. 1c–e), these results suggest that the 0–8 cm community might be more resilient to disturbances. Analysing the density, mean clustering coefficient and mean path length of the 14–20 cm co-occurrence networks indicated a higher probability of microbial interactions and connections.

**Fig. 4.**
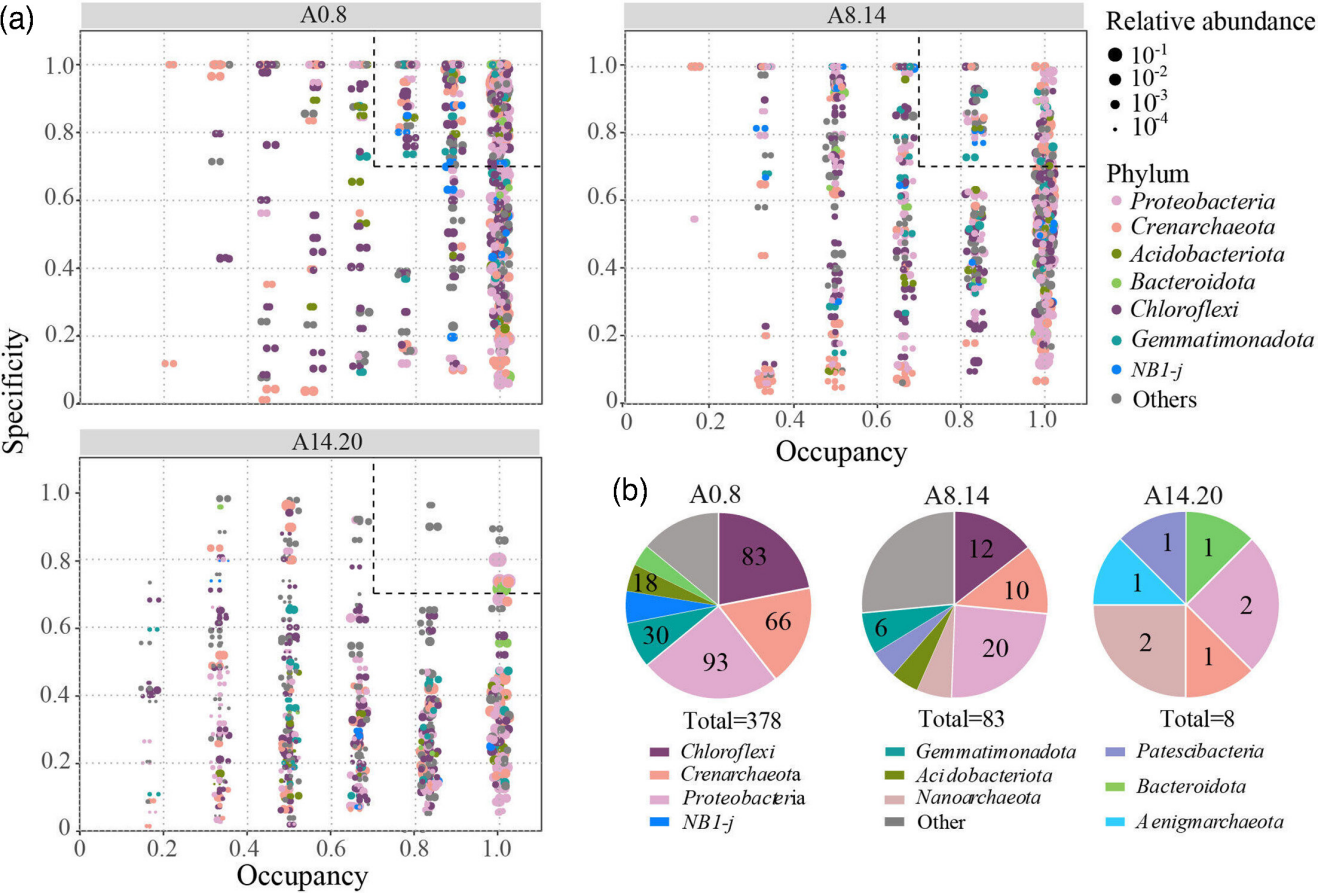
Specialized species based on SPEC-OCCU plots and microbial composition at the phylum level. (**a**) SPEC-OCCU plots of the top 500 ASVs in the three groups. The x-axis reflects the ASV distribution across all samples (occupancy), whereas the y-axis indicates its uniqueness, showing whether the ASV is exclusive to certain samples. (**b**) Composition of specialized species in three groups. ASVs with both specificity and occupancy ≥0.7 are considered specialized species (dashed box).

Keystone ASVs were identified according to within-module connectivity (Zi)-among-module connectivity (Pi) relationships and SPEC-OCCU analysis. In the Zi-Pi plots, 19, 10 and 11 ASVs were classified as module hubs, showing strong connections to nodes within their respective modules (Fig. 3d, f and h). The majority of ASVs were not annotated to the genus and species levels (Table S3), which might be analogous to potential keystone species in the microbial community, indicating the presence of a large number of potentially novel organisms. In SPEC-OCCU plots, the ASV occupancy was relatively uniform in the A0.8 group, while it was more varied in the A8.14 and A14.20 groups (Fig. 4a). A total of 37,883 and 8 specific ASVs were identified based on their specificity and occupancy (≥0.7) in the 0–8, 8–14 and 14–20 cm groups, respectively (Fig. 4b). The identified ASVs were primarily from the *Chloroflexi*, *Proteobacteria* and *Crenarchaeota* phyla. Additionally, NB1-j (five ASVs) was found exclusively in the 0–8 cm group, while *Aenigmarchaeota* (one ASV) was only found in the 14–20 cm group.

### Effects of biogeochemical factors on microbial community

**Fig. 5.**
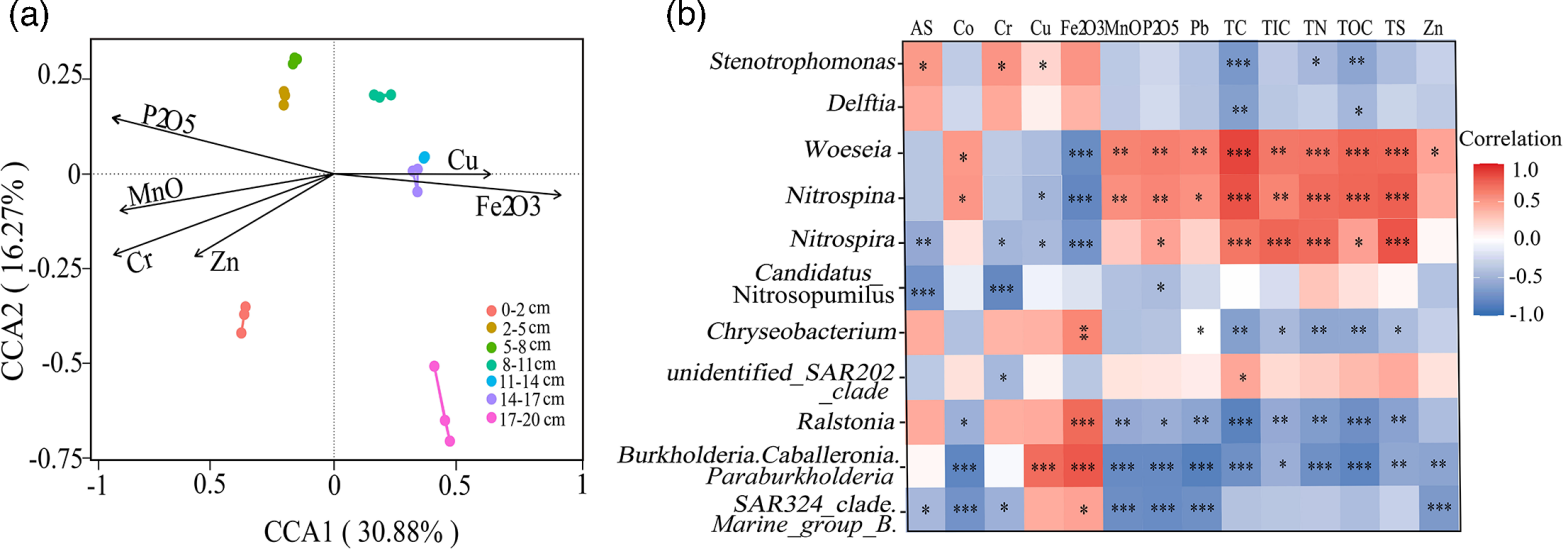
Correlation analysis between microbial communities and environmental factors. (a) CCA analysis of various environmental factors. (b) Heatmap diagram of Spearman's correlation between environmental factors and dominant genera. The correlation was illustrated using different colours, with asterisks indicating significance (*p < 0.05, ** *p* <0.01, *** *p* < 0.001).

To further elucidate the mechanisms of community composition, the co-relationship among samples and environmental variables was assessed using CCA plots (Fig. 5a). The first two axes of the CCA plot explained the variances for 30.88% and 16.27% in all samples and showed that the effects of chemical variables (Fe_2_O_3_, Cu, P_2_O_5_, MnO, Cr and Zn) on samples vary with depth (Fig. 5a). Microbial distribution was majorly affected by P_2_O_5_, Fe_2_O_3_, MnO and heavy metals such as Cr, Zn and Cu. The correlations between environmental factors and microbial taxa showed Fe_2_O_3_ and As showed significant negative correlations with *Nitrospirota*, *Gemmatimonadota* and NB1-j (Fig. S3). *Proteobacteria*, the most abundant phylum of bacteria, only had positive correlations with As and Cr. *Stenotrophomonas*, belonging to *Proteobacteria*, had positive correlations with As, Cr and Cu.

In addition, we also found that *Woeseia*, *Nitrospina*s and *Nitrospira* showed significant positive correlations with TC, TIC, TN, TOC and TS. For example, *Nitrospina*s and *Nitrospira,* belonging to *Nitrospinota*, had significant positive correlations with TN (Fig. 5b). The TN concentration was greatest in the top layer, then decreased in the middle layer and was lowest in the bottom layer. *Nitrospira* exhibited the highest relative abundance in the surface layer, indicating that the reduction of TN content might lead to a decline in the abundance of *Nitrospira. Woeseia* is a globally distributed facultative chemolithoautotroph that utilizes inorganic sulphur and hydrogen compounds, commonly found in marine sediments [46,47]. Similarly, the abundance of *Woeseia* in the surface, middle and bottom layers showed a pattern similar to that of TC, TIC, TN, TOC and TS concentrations, with highest in the surface layer, followed by the middle layer and the lowest in the bottom layer. These findings support the influence of metal elements and nutrient cycling on the species abundance.

### Metabolic potential of subseafloor prokaryotes

**Fig. 6.**
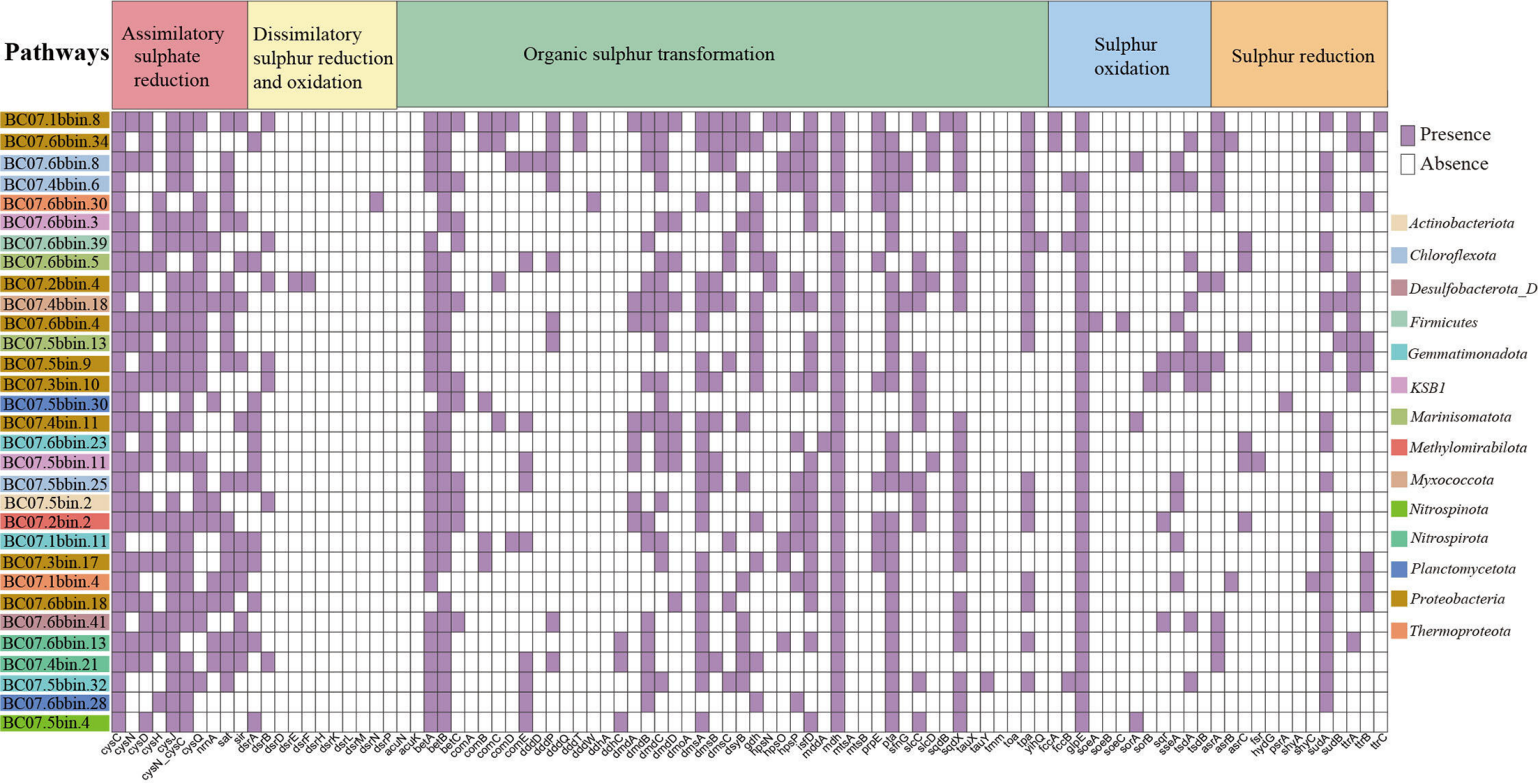
The presence of key genes involved in various S metabolism pathways was observed in high-quality bins. MAGs with completeness > 90% and contamination <5% are defined as high-quality bins.

The dominant microbial genomes and important gene sequences encoding metabolic pathways were obtained through metagenomic sequencing. We analysed the gene annotated ratio associated with N, S, C, Fe, As and plastic cycling metabolic pathways across surface (0–8 cm), middle (8–14 cm) and bottom layers (14–20 cm). The results showed that S metabolism pathways had the highest number of annotated genes across all layers, followed by P, C, As and N metabolism pathways (Fig. S4A). Statistical analysis of the gene annotation counts for sulphur metabolism pathways revealed that sulphur transformation pathways had the highest number of annotated genes, followed by sulphur oxidation pathways and dissimilatory sulphate reduction and sulphur oxidation (Fig. S4B). Among the 122 metagenome-assembled genomes (MAGs) obtained, consisting of 113 bacterial and 9 archaeal genomes, 100 MAGs were potential new species lacking species-level annotation (Tables S4 and 5). *Proteobacteria* (13 MAGs) was the dominant bacterial phylum among these bins annotation, which is consistent with the results obtained from the 16S rRNA gene amplicon analysis. We obtained 27 MAG high-quality bins with completeness >90% and contamination <5%. We found that 27 MAGs were primarily responsible for assimilatory sulphate reduction and organic sulphur transformation pathways. These findings highlight the central role of sulphur metabolism, especially assimilatory sulphate reduction and organic sulphur transformation, in deep-sea microbial communities (Fig. 6).

**Fig. 7.**
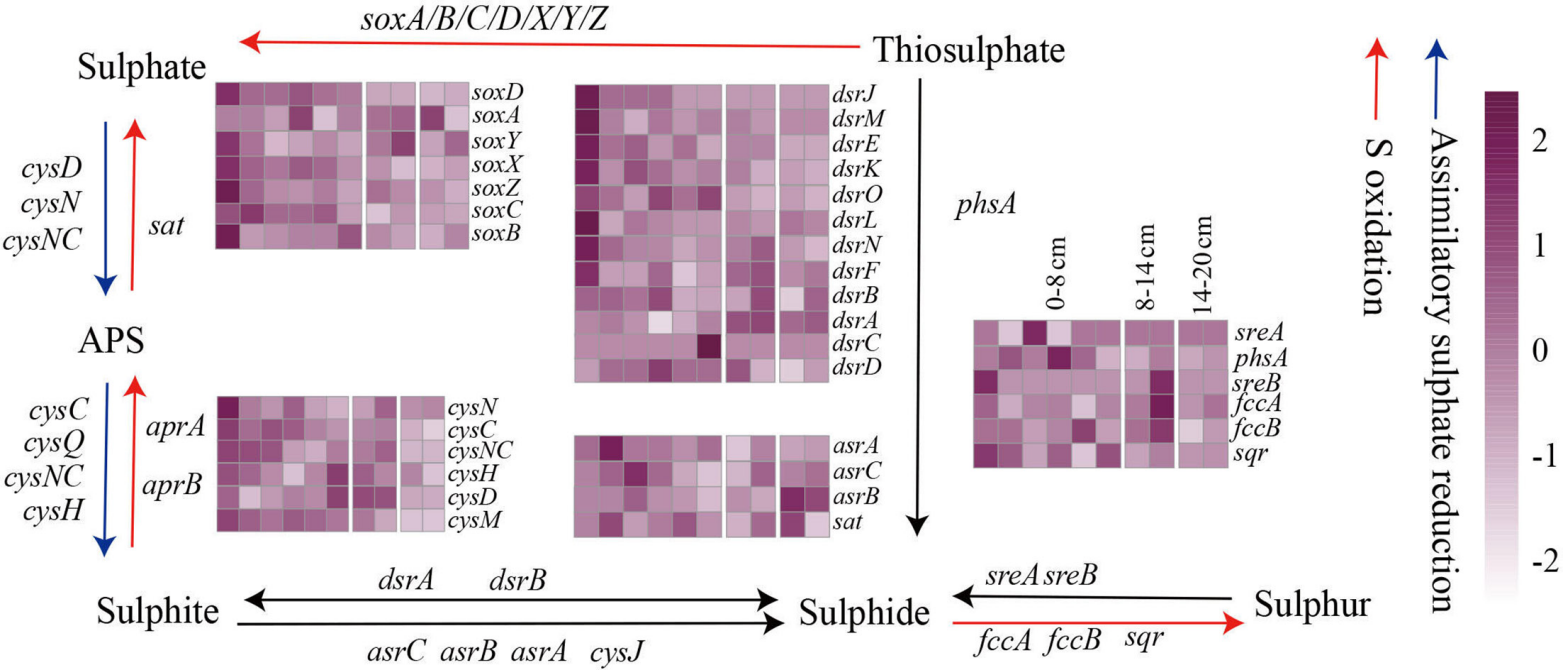
Heatmaps displaying the Z-score normalized relative abundances of key functional genes involved in S cycling.

To resolve the depth-specific roles of sulphur metabolism, we analysed key gene distributions. In our study, assimilatory sulphate reduction genes *cysN*, *cysC*, *cysNC*, *cysD* and *cysH* showed significantly higher abundances in the 0–8 and 8–14 cm layers compared to the 14–20 cm layer (Fig. 7), suggesting that microbes in the surface are more capable of assimilatory sulphate reduction than deeper layers. The *dsr* gene family *dsrA* and *dsrB* encode the alpha- and beta-subunits of DSR, respectively. They are involved in the process of dissimilatory sulphate reduction. In middle (8–14 cm) and bottom layers (14–20 cm), dissimilatory sulphate reduction genes *dsrA* and *dsrB* had higher gene abundance than in the surface layer (0–8 cm). In contrast, the *dsrM*, *dsrJ*, *dsrK*, *dsrP*, *dsrE*, *dsrL*, *dsrN* and *dsrF* showed elevated gene abundance in the surface layer, indicating active sulphur-sulphide interconversion. Additionally, S oxidation is the primary energy source for many chemolithoautotrophic micro-organisms. Through S oxidation, these micro-organisms obtain energy to sustain their life activities. In our research, the heatmap of sulphite oxidation genes *soxB/C/D/X/Y/Z* showed higher gene abundance in the surface layer compared to other depths (Fig. 7), indicating active sulphur metabolism potential in surface sediments. We speculated that micro-organisms in the surface layer may obtain energy for their life activities through sulphur oxidation.

## Discussion

Investigating the diversity and vertical distribution of microbes in deep-sea sediments is crucial to improve our understanding of their ecological roles in facilitating metabolic activities [48,49]. The Magellan seamount chain, located in the western Pacific, comprises top flat seamounts ranging in depth from 1,500 to 6,000 m. These seamounts are known for their rich biodiversity and support a wide variety of ecosystems [50,51]. The 16S rRNA gene amplification sequencing and metagenomics are widely used to reveal the diversity of uncultured microorganisms and microbial metabolic capacity in deep-sea sediments [52,53]. In our research, the three sediment layers (0–8, 8–14 and 14–20 cm) exhibited distinct microbial community diversities, as shown in Fig. 1. The richness and diversity of microbial communities at sediment depths of 8–14 and 14–20 cm were significantly lower than those at 0–8 cm (Fig. 2c–e), indicating that microbial diversity and abundance decreased dramatically with sediment depth at the Magellan seamount. This sharp decrease in prokaryotic *α*-diversity from surface to deeper layers was consistent with previous studies in deep marine environments [49,54]. Previous studies have found that the declines in the microbial diversity and abundance are associated with oxygen levels, nutrient availability and their distribution within sediment layers [55,57]. A recent study provided evidence that polymetallic nodules may act as a "geo-battery" through seawater electrolysis, generating oxygen (termed dark oxygen production) in the dark, abyssal environment of the Pacific Ocean's Clarion-Clipperton Zone (CCZ) [58]. The oxygen levels decrease with sediment depth [56,59], and this oxygen depletion is associated with reduced microbial diversity in deeper layers [55,59, 60]. *Nitrosopumilus*, a group of aerobic archaea, utilizes oxygen as an electron acceptor to oxidize ammonia (NH₃^+^) to nitrite (NO₂⁻) [61]. In our results, the relative abundance of *Candidatus_Nitrosopumilus* (belonging to the phylum *Thaumarchaeota*) gradually decreased with depth, reaching its lowest level in the 14–20 cm sediment samples (Fig. S2). Therefore, this change may be linked to the decline in oxygen availability at greater depths.

Previous research in Nazimov guyots of Magellan seamounts demonstrated that *Proteobacteria* was the most prevalent populations at the phylum level, while the abundance of *Chloroflexi* increased in the deep layer. The overall distribution of *Chloroflexi* seems to be influenced by available nitrogen [17]. Our results also found that *Proteobacteria* was the most abundant phylum, but notably, the abundance of *Chloroflexi* decreased in deeper layers (Fig. 2a). *Chloroflexi* exhibited significant correlations with TC, TIC, TN, TOC and TS (Fig. S2). Those results indicated that this pattern may be linked to the reduction of these environmental factors with depth. Additionally, the other micro-organisms with high relative abundances in our research differed significantly from previous findings. Furthermore, the dominant archaeal phylum differed between studies: *Crenarchaeota* in our sediments (Fig. 2) versus *Thaumarchaeota* in Nazimov Guyots [17]. We hypothesize that such divergence may stem from distinct metal availability patterns. Specifically, our data revealed significant depth-dependent variations in Co and Zn contents. This metal gradient is particularly relevant given known metalloenzyme requirements in archaea. Zinc ions (Zn^2+^) serve as the natural cofactor bound to the active form of the archaeon *Pyrobaculum aerophilum* alcohol dehydrogenase. Investigations of metal ion functionality demonstrate that cobalt (Co) can be structurally incorporated into the enzyme, acting as a functional substitute for zinc [62]. Prior studies indicate that *Crenarchaeota* utilize divalent metals (e.g. Mn^2+^/Mg^2+^) for essential enzymes like pyruvate kinase, which exhibits dual-metal affinity [63]. Importantly, the observed depth-dependent metal gradients (Co and Zn) suggest microbial communities in these seamounts are finely adapted to localized geochemical conditions. This specialization implies that physical disturbances, such as those caused by deep-sea mining activities targeting polymetallic nodules, could disrupt these delicate metal-microbe relationships. Resuspension of metal-rich sediments during mining operations may alter metal bioavailability and redox conditions, potentially eliminating niche-adapted communities and reducing functional redundancy. However, whether Co/Zn similarly influence their metabolism remains unexplored, warranting future investigation.

Co-occurrence networks have been effectively used to reveal specific yet subtle relationships and to infer complex interactions within microbial communities in marine and other environments [64,66]. Co-occurrence patterns in microbial communities are closely related to species diversity, abundance and interactions [67]. In this study, network topology statistics revealed that the overall network had higher values for nodes, edges, average degree and density compared to individual co-occurrence networks, indicating that the overall network is the most robust (Table S2). Microbial co-occurrence patterns at different depths showed more complex structures in sediments at a depth of 0–8 cm than in deeper layers (Fig. 3), which might be linked to the highest microbial diversity in surface layer. The diversity of microbial community tends to decrease with increasing sediment depth, leading to fewer interactions and connections among microbes.

Deep-sea microbial communities may be influenced by geochemical conditions that vary according to geographic features [68,69]. In our study, the concentrations of Fe₂O₃ and As were higher in the bottom layer than in the middle and surface layers, while the concentrations of MnO, Co, Zn, Pb and nutrients such as TC, TOC, TIC, TN and TS were highest in the surface layer, followed by the middle and bottom layers. The CCA plot indicated that microbial distribution was significantly influenced by P_2_O_5_, Fe_2_O_3_, MnO and heavy metals such as Cr, Zn and Cu (Fig. 5a). Our results revealed that *Stenotrophomonas* was the dominant genus (Fig. S2), and they exhibited significant positive correlations with As, Cr and Cu (Fig. 5b). This finding aligns with its known metabolic versatility in metal-rich environments. As a facultative anaerobe, *Stenotrophomonas* can utilize multiple electron acceptors (e.g. Fe³^+^ and Cr⁶^+^) under fluctuating redox conditions [70], which likely explains its dominance in the bottom layer, where elevated concentrations of Fe₂O₃ and As were observed in our study. This metabolic adaptability highlights the potential for some microbial groups to withstand perturbations like metal mobilization during mining. However, the dominance of specific metal-tolerant taxa following disturbance would likely come at the expense of overall diversity and the loss of specialized functions performed by sensitive groups.

Nutrients (TN, TS and TC) in the samples decrease with changes in sediment profiles, which may lead to changes in microbial richness and diversity with sediment depth [7]. Our results indicated that TN, TS, TC, TOC and TIC concentrations gradually declined with increasing sediment depth, and microbial diversity and abundance also decreased with increasing depth (Fig. S1). *Chloroflexi* is a highly diverse group. Members of the phylum *Chloroflexi* exhibit metabolic versatility in both carbon and sulphur cycling including assimilatory sulphate reduction and fixing inorganic CO₂ [70,72]. *Phototrophicus methaneseepsis ZRK33*, belonging to *Chloroflexi*, converts sulphate or thiosulphate into cysteine through assimilatory sulphate reduction, thereby driving energy cycling through other metabolic pathways [73]. Organic sulphur compounds are more easily adsorbed or incorporated into bioclastic material and fixed. The abundance of *Chloroflexi* decreased as depth increased (Fig. 2), and there was a remarkable positive correlation between *Chloroflexi* and TS (Fig. S3), which is consistent with the variations in TS concentrations at different depths. We hypothesized that the decline in *Chloroflexi* abundance may weaken their sulphur retention capacity, leading to the reduction in TS content in sediments. In addition, ammonia-oxidizing bacteria *Nitrospina* and *Nitrospira* showed significant correlations with TN, TS and TC (Fig. 5b). They had the highest relative abundance in the surface layer, followed by the middle and the bottom layers, which was consistent with the differences in TN, TS and TC levels. Therefore, these results suggested that heavy metals and nutrients were the key factors driving the distribution change of prokaryotes in the sediments.

Microbial functional diversity plays critical roles in maintaining ecosystem stability by driving key biogeochemical cycles (e.g. carbon, sulphur and nitrogen). Sulphur cycling represents a cornerstone of marine sediment biogeochemistry, driven primarily by the interplay between sulphate-reducing bacteria (SRB) and SOB. SRB utilized sulphate (SO₄²⁻) as a terminal electron acceptor, while SOB oxidize reduced sulphur species (e.g. sulphide and thiosulphate), collectively sustaining sulphur transformations essential for sediment redox dynamics [74,75]. Our metagenomic data revealed that S metabolism pathways had the highest number of annotated genes across all layers (Fig. S4A). Further statistics revealed that the number of genes annotated to the sulphur transformation pathway was the highest, followed closely by sulphur oxidation and dissimilatory sulphate reduction and oxidation (Fig. 6 and S4B). This genomic potential underscores the central role of sulphur cycling in this seamount ecosystem and its contribution to broader ocean biogeochemistry. In S cycling, thiosulphate, an intermediate sulphur compound, serves as a vital link in the network of numerous sulphur metabolic pathways [76]. The *SOX* genes (*soxA*, *soxB*, *soxC*, *soxD* and *soxZY*) are involved in thiosulphate oxidation through the SOX pathway [77,78]. The abundance of the *soxA*, *soxB*, *soxC*, *soxD*, *soxZ* and *soxY* decreased from the surface to the bottom layer, indicating a decline likely due to decreasing oxygen content with depth (Fig. 7). SOX genes were primarily affiliated with chemolithoautotrophic sulphur-oxidizing genera within *Proteobacteria*, including *Thiobacillus* (*γ*-proteobacteria) and *Sulfurovum* (*ε*-proteobacteria). Assimilatory sulphate reduction *CysN*, *CysC*, *CysNC* and *CysM* in the surface sediment (0–2 cm) had higher gene abundance than other sediments, indicating the higher demand for reduced sulphur for the formation of organic sulphur compounds. Heterotrophic *Bacteroidetes* and *Gammaproteobacteria* in surface sediments showed multiple assimilatory sulphate reduction genes. Taken together, a possible reason is that the oxygen in the upper water was sufficient and the sulphur oxidation process was more frequent. Deep-sea mining activities, targeting nodules often found within these critical surface and subsurface layers (0–20 cm), would directly disrupt these stratified microbial communities and their associated biogeochemical functions. Sediment plumes generated by mining could smother communities, alter redox gradients essential for sulphur cycling and disperse metal-rich particles far beyond the mining site [79]. Recovery of the intricate cycling networks, especially the slow-growing, depth-specialized communities, could take centuries or longer given the low energy flux in the deep sea [80]. Therefore, conservation policies for seamounts and nodule fields must prioritize the protection of these microbial ecosystem engineers. Mining regulations need to incorporate microbial diversity and function as key indicators of ecosystem health and establish stringent thresholds for acceptable disturbance to preserve these biogeochemical services. Future research should explicitly quantify the contribution of seamount microbial sulphur cycling to regional and global budgets and model the long-term functional consequences of community disruption caused by resource extraction.

## Conclusion

Understanding vertical variations in microbial community structure, diversity, interactions and environmental adaptations on seamounts is crucial for exploring how environmental factors and biogeochemical cycling shape the subsurface biosphere in seafloor sediments. Our results highlight the key role of depth in shaping prokaryotic distribution, with microbial communities in surface sediments (0–8 cm) exhibiting higher diversity, abundance and complexity than those found in deeper layers (8–20 cm). Additionally, microbial communities in surface sediments were more resilient to disturbance, while those at greater depths displayed stronger bacterial interactions and connectivity. The factors TN, TS and TC are critical drivers of the microbial community, as reflected in their significant correlations with various taxa in the Magellan seamount sediments. Metagenomic analyses revealed a higher activity of assimilatory sulphate reduction and sulphate oxidation in the surface sediments compared to deeper layers, with a significant abundance of sulphur cycle-related genes (e.g. *SOX*, *CysN*, *CysC*, *CysNC* and *CysM*) in the surface microbial communities. Our findings enhance the understanding of microbial diversity and metabolic processes in deep-sea sediments beneath polymetallic nodules, offering valuable insights for future deep-sea mining operations. Critically, the observed depth-stratified microbial communities and their distinct metabolic potentials, particularly regarding sulphur cycling, underscore the complex interplay between geochemistry and microbiology in shaping deep-sea benthic ecosystems and regulating key biogeochemical fluxes in these vast, yet fragile environments. Future research should focus on elucidating the *in situ* activity of these key sulphur-cycling pathways through metatranscriptomic or proteomic analyses and experimentally validate the predicted microbial interactions (e.g. syntrophy) using targeted cultivation approaches to gain deeper mechanistic insights into community functioning and resilience under changing environmental conditions.

## Supplementary material

10.1099/mgen.0.001493 Uncited Supplementary Material 1.

10.1099/mgen.0.001493 Uncited Supplementary Material 2.

## Abbreviations

CCA: canonical correspondence analysis
MAGs: metagenome-assembled genomes
PCoA: principal coordinate analysis
SOB: sulphur-oxidizing bacteria
SRB: sulphate-reducing bacteria
TC: total carbon
TIC: total inorganic carbon
TN: total nitrogen
TOC: total organic carbon
TS: total sulphur
