# Supplementary material for: Vertically stratified microbial diversity and keystone species driving element cycling in the Magellan seamount sediments
Source: Microb Genom. 2025 Dec 5;11(12):001493. doi: 10.1099/mgen.0.001493 (PMC13293335; doi:10.1099/mgen.0.001493)
Supplement: Uncited Supplementary Material 1. [file mgen-11-01493-s001.pdf]

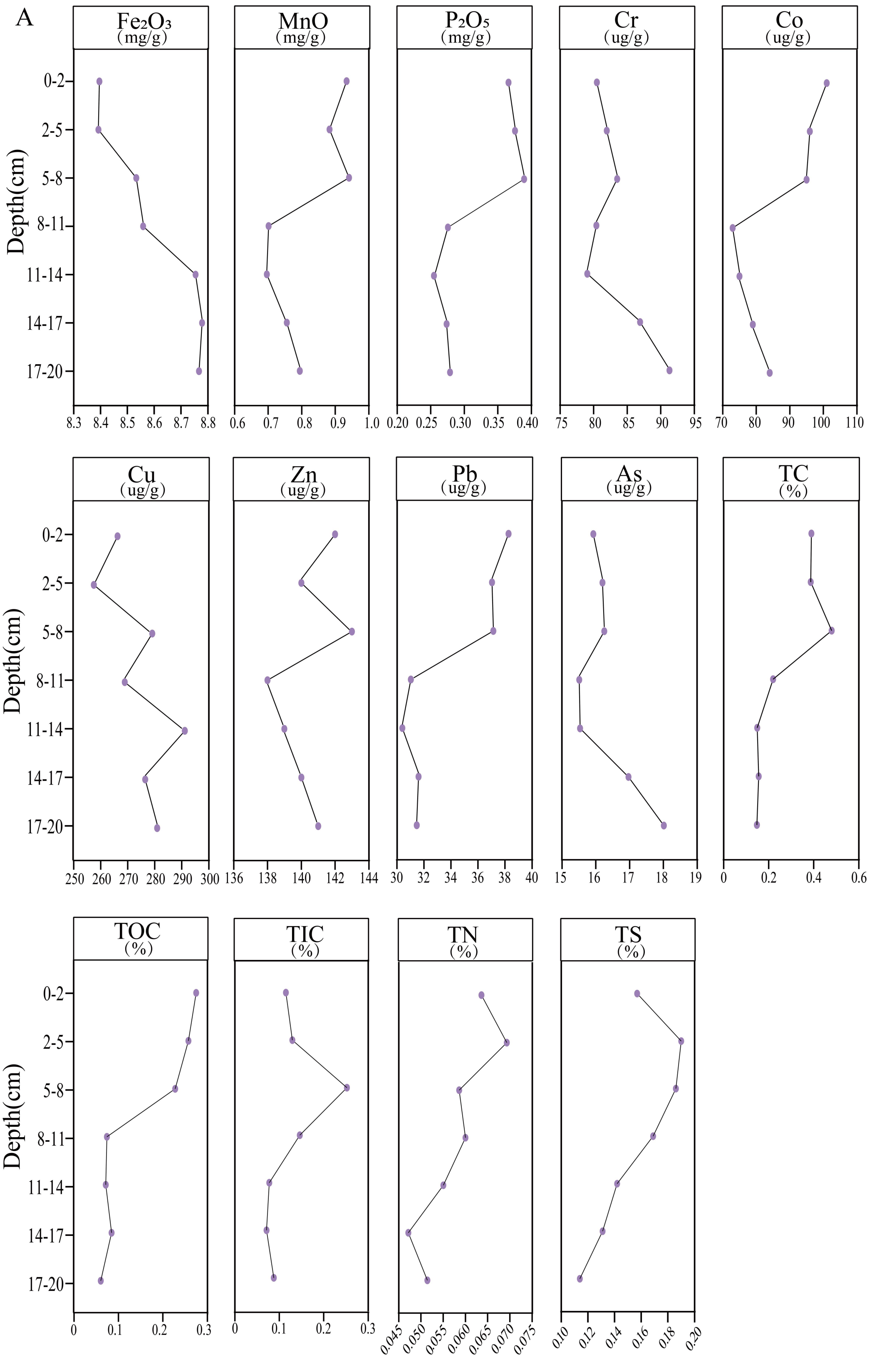

Fig.S1. Environmental characterization of sediments at different depths. Abbreviations: TN, total nitrogen; TC, total carbon; TIC, total inorganic carbon; TOC, total organic carbon; TS, total sulfur. The cmbsf means cm below the seafloor.

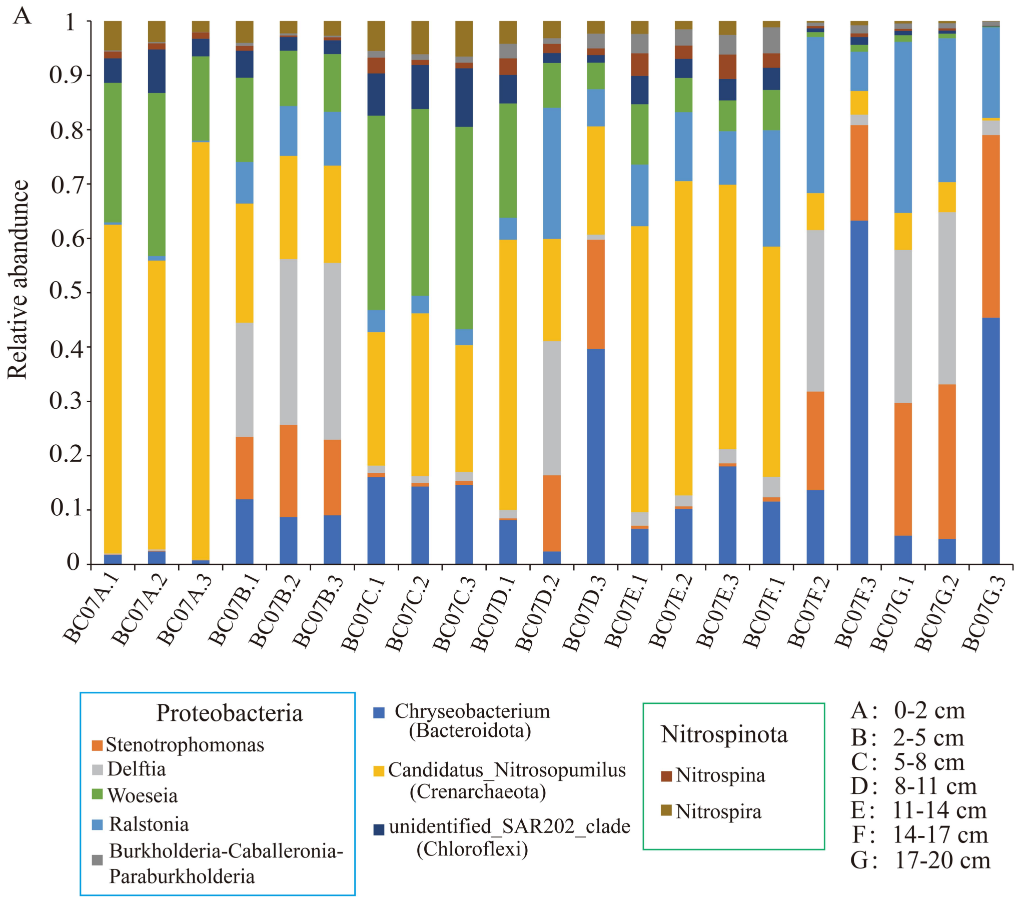

Fig.S2. Relative abundance of dominant microorganisms at the genus level in BC07 sediment.

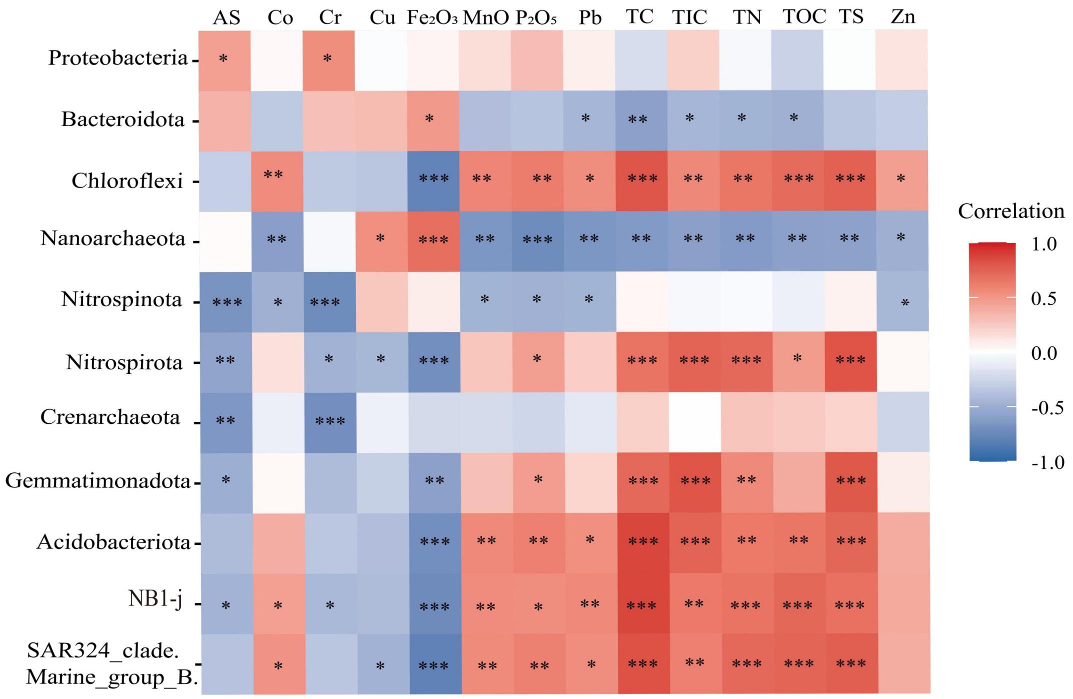

Fig.S3. Heatmap diagram of Spearman's correlation between environmental factors and dominant phyla.

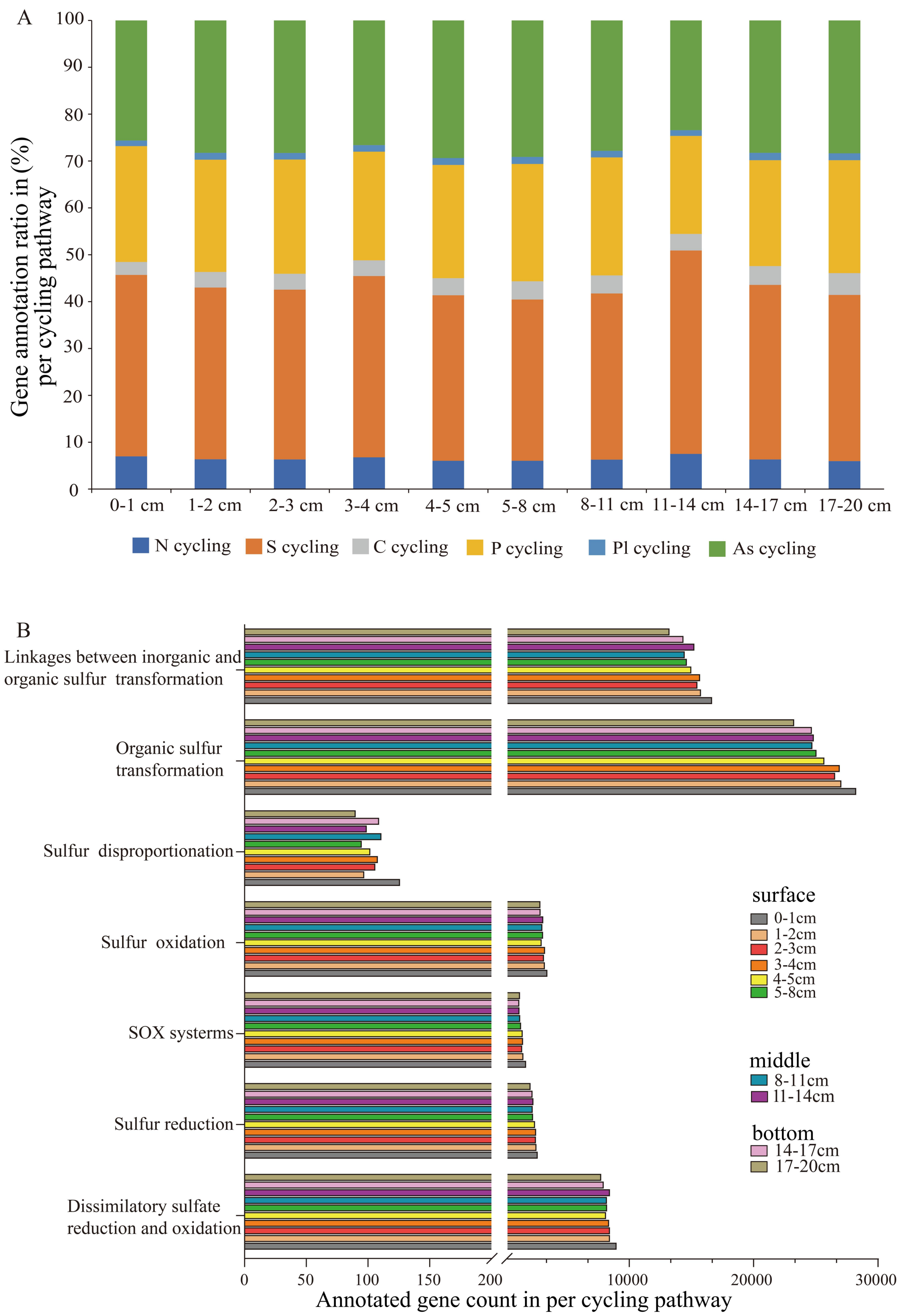

Fig.S4. The relative abundances of different metabolic pathways. (A) The relative abundances of N, S, P, C, Fe, and Plastic cycling pathways (B) Sulfur metabolism pathway abundances of the samples.
